# Association of Decreased Bone Density and Hyperlipidemia in a Taiwanese Older Adult Population

**DOI:** 10.1210/jendso/bvae035

**Published:** 2024-03-05

**Authors:** Hui-Ming Chen, Chung-Yuan Hsu, Bo-Lin Pan, Chih-Fang Huang, Chao-Tung Chen, Hung-Yi Chuang, Chih-Hung Lee

**Affiliations:** Department of Family Medicine and Occupational Medicine, Kaohsiung Chang Gung Memorial Hospital, Kaohsiung, 833401, Taiwan; Division of Rheumatology, Allergy, and Immunology, Department of Internal Medicine, Kaohsiung Chang Gung Memorial Hospital, Kaohsiung, 833401, Taiwan; School of Medicine, College of Medicine, Chang Gung University, Taoyuan, 333323, Taiwan; Department of Family Medicine and Occupational Medicine, Kaohsiung Chang Gung Memorial Hospital, Kaohsiung, 833401, Taiwan; Department of Family Medicine, Kaohsiung Municipal Feng-Shan Hospital, Kaohsiung, 830025, Taiwan; Department of Long-Term Care and Management, Chung Hwa University of Medical Technology, Tainan, 717302, Taiwan; Department of Family Medicine, Chang Gung Memorial Hospital, Chiayi, 613016, Taiwan; Department of Public Health, Kaohsiung Medical University, Kaohsiung, 807378, Taiwan; Department of Environmental and Occupational Medicine, Kaohsiung Medical University Hospital, Kaohsiung, 807377, Taiwan; School of Medicine, College of Medicine, Chang Gung University, Taoyuan, 333323, Taiwan; Department of Dermatology, Kaohsiung Chang Gung Memorial Hospital, Kaohsiung, 833401, Taiwan

**Keywords:** osteopenia, osteoporosis, lipid profile, dyslipidemia

## Abstract

**Objective:**

This study aimed to determine if a combination of 2 abnormal lipid profiles revealed a stronger association with low bone mass than a single blood lipid abnormality alone.

**Methods:**

This study enrolled 1373 participants who had received a dual-energy x-ray absorptiometry scan from January 2016 to December 2016 in a medical center in southern Taiwan. Logistic regression was used to examine association between lipid profiles and osteopenia or osteoporosis after adjusting for covariates.

**Results:**

Compared to people with total cholesterol (TC) < 200 mg/dL, those with TC ≥ 240 mg/dL tended to have osteopenia or osteoporosis (OR 2.61; 95% CI, 1.44-4.71). Compared to people with low-density lipoprotein cholesterol (LDL-C) < 130 mg/dL, those with LDL-C ≥ 160 mg/dL tended to develop osteopenia or osteoporosis (OR 2.13; 95% CI, 1.21-3.74). The association of increased triglyceride and decreased bone mass was similar, although not statistically significant. Those with the combination of TG ≥ 200 mg/dL and TC ≥ 240 mg/dL had a stronger tendency to have osteopenia or osteoporosis (OR 3.51; 95% CI, 1.11-11.13) than people with only one blood lipid abnormality. Similarly, people with TG ≥ 200 mg/dL and LDL-C ≥ 160 mg/dL had a stronger tendency to have osteopenia or osteoporosis (OR 9.31; 95% CI, 1.15-75.42) than people with only one blood lipid abnormality, after adjustment for the same covariates.

**Conclusion:**

Blood levels of TC, LDL-C, and TG were associated with osteopenia or osteoporosis. Results indicate that individuals aged older than 50 years with abnormal lipid profiles should be urged to participate in a bone density survey to exclude osteopenia or osteoporosis.

Osteoporosis and osteopenia are severe public health issues that affect over 200 million individuals globally [1]. The country-specific prevalence of osteoporosis at the total hip or hip/spine ranges from 9% to 38% for women and 1% to 8% for males in 9 industrialized nations in North America, Europe, Japan, and Australia [2], although the global prevalence of osteoporosis varies significantly between continents and countries [3]. In Taiwan, a nationwide survey found that the prevalence of osteoporosis increased from 17.4% in 2001 to 25.0% in 2011 [4]. Patients with osteoporosis might become immobile and require additional care due to fractures in the spine and hips [5]. Although the pathogenesis of osteoporosis remains unclear, several risk factors have been documented in association with osteoporosis, including advancing age, gender, the presence of peptic ulcers, and smoking [5-9]. Interestingly, a recent Finland cohort study based on 1545 patients supported the osteoporosis-atherosclerosis comorbidity hypothesis [8].

Dyslipidemia is a key risk factor for atherosclerosis that affects various sizes of vessels, consequently causing ischemia in the brain, heart, and legs [9]. Increased blood levels of total cholesterol (TC), low-density lipoprotein cholesterol (LDL-C) and triglycerides (TG) as well as low blood levels of high-density lipoprotein cholesterol (HDL-C) are associated with increased risks of cerebral vascular disease in both randomized trials [10] and large observational studies [11].

These oxidized lipids are not only risk factors for atherosclerosis but may also contribute to osteoporosis; they have been shown to decrease osteoblastic development and reduce bone marrow density. Concurrently, these oxidized lipids can cause atherosclerosis by induction of the osteoblasts in the vessel wall to mineralize and differentiate [12-14].

Although animal studies have indicated that an atherogenic high-fat diet reduces bone mineral density (BMD) [15], the association between lipid profile and osteoporosis remains undetermined in human research [16-20]. Several investigations have revealed that lipid profiles and BMD had no significant associations [16-18], although on the other hand, increased blood levels of LDL and TC were discovered to be associated with osteoporosis by Orozco [19] and Kan et al [20]. One meta-analysis that included 12 studies (12 395 subjects) found that only HDL-C was elevated in patients with osteoporosis [21]; however, another meta-analysis revealed that there was no statistical difference in LDL and TG between osteoporosis and normal density groups, albeit the serum levels of HDL and TC were higher in postmenopausal patients with osteoporosis [22].

To date, the associations between lipids with osteoporosis are debatable and few similar studies have been conducted regarding this issue in Taiwan. Loke et al found that HDL-C was positively associated with BMD in women, but negatively associated with BMD in men [23], so gender effect could be a potential confounding factor that might affect BMD and lipid profiles. Accordingly, we designed a retrospective cross-sectional study with adjustment for gender, age, and body mass index (BMI), to (i) examine the associations of elevated TC, LDL-C, HDL-C, or TG with osteopenia or osteoporosis; (ii) investigate the gender effect on lipid profiles and osteopenia or osteoporosis; and (iii) determine if a combination of 2 abnormal lipid profiles had a higher association with osteopenia or osteoporosis.

## Methods

### Subjects

**Figure 1.**
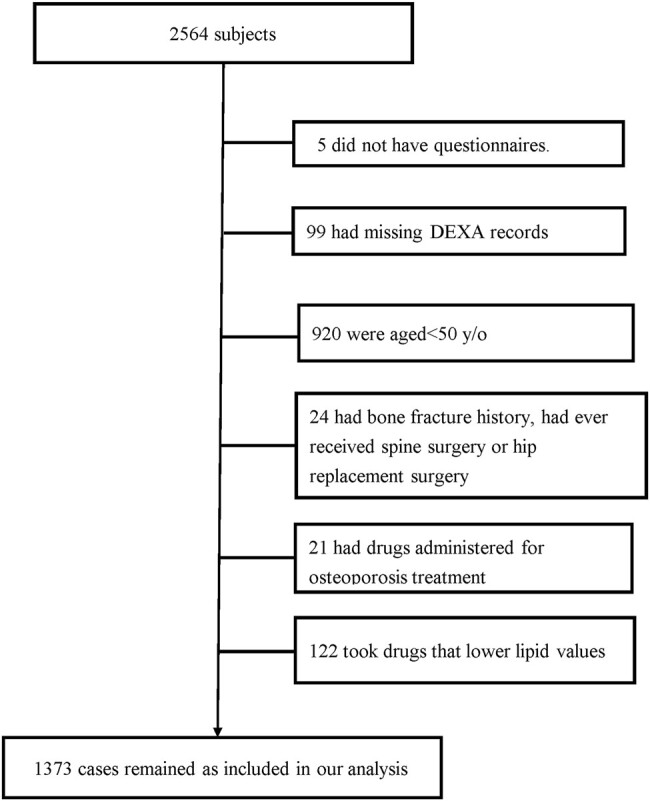
Study flowchart.

Data from a health examination database in Kaohsiung Chang Gung medical center in southern Taiwan were used in this retrospective cross-sectional study. Our hospital provides a range of health examinations and procedures, including physical examination, blood biochemical analysis, dual-energy x-ray absorptiometry (DXA) BMD measurement, and gastroscopy. Between January 2016 and December 2016, 2564 participants who had undergone self-paid health examinations with a DXA scan were included. Most of the participants had no symptoms and did not regularly consume alcohol. To assure the healthy and optimal condition of study subjects, those who did not have questionnaires on file (n = 5), or those who were aged < 50 years (n = 920), with missing DXA records (n = 99), with bone fracture history, had ever received spine surgery or hip replacement surgery (n = 24), under drug treatment for osteoporosis (n = 21), or presently took drugs that lowered lipid values (n = 122) were excluded. Finally, a total of 1373 eligible subjects were included in this study (Fig. 1). Written consent was waived by the institutional review board (IRB# 202200344B0C601).

### Determination of Bone Mineral Density

BMD was measured by a DXA scan (Lunar iDXA, GE Healthcare), with the least significant change of the study DXA machine being 0.028 g/cm^2^ in the spine, 0.039 g/cm^2^ in the hip, and 0.055 g/cm^2^ in the forearm, with these densities of the non-dominant forearm, femur, and lumbar spine regions being measured by a specialist. The T-score is the number of standard deviations a measurement deviates from the mean for a healthy young adult population aged from 20 to 29 years, and the Caucasian (not race-adjusted) female normative database for women and men of all ethnic groups was used as the reference database. According to World Health Organization guidelines, the lowest T-score between the 3 sites was divided into 3 groups: normal (T-score equal to or more than −1.0), osteopenia (T-score < −1.0 and greater than −2.5), and osteoporosis (T-score equal to or less than −2.5) [24]. Osteoporosis and osteopenia were defined as decreased BMD in the current investigation.

### Measurements of Lipid Profile and Covariates

All of the subjects had their blood biochemically analyzed. After fasting for 12 hours, blood samples were collected from a peripheral vein and centrifuged at 4 °C. Biochemical analysis included fasting blood glucose, uric acid, liver function, and the lipid profile (TC, LDL-C, TG, HDL-C). Anthropometric status, BMI, and waist circumference (WC) were also measured for all subjects, while a stadiometer with a balance beam scale was used to determine height and weight. The tape was used to measure the circumference of the waist with weight/height squared (kg/m^2^) being used to calculate BMI. Systolic and diastolic blood pressure was measured twice by a well-trained nurse using a standard sphygmomanometer (and averaged), while the diagnosis of peptic ulcer was determined by gastroscopy.

### Statistical Methods

The characteristics of subjects were compared by χ^2^-test for the categorical variables and Student *t*-test or analysis of variance (ANOVA) for the continuous variables. Simple and multiple logistic regressions were used to examine the association between lipid profile and osteopenia or osteoporosis after adjustment for age, gender, BMI, systolic blood pressure (SBP), diastolic blood pressure (DBP), fasting blood sugar, uric acid, HDL, alanine aminotransferase (ALT), and gastric ulcer. According to the National Cholesterol Education Program (NCEP) Adult Treatment Panel III (ATPIII) [25], TG concentration was categorized into 3 groups: normal (< 150 mg/dL), borderline high (150∼199 mg/dL) and high (≥ 200 mg/dL). TC and LDL-C were also categorized into 3 groups, respectively: normal/near normal (< 200 mg/dL, < 130 mg/dL), borderline high (200∼239 mg/dL, 130-159 mg/dL) and high (≥ 240 mg/dL, ≥ 160 mg/dL). To measure the impact of gender on the associations between lipid profiles and lower BMD, we conducted a subgroup analysis stratified by gender, while multiple regression analysis with gender stratification was used to investigate the associations between the atherogenic indexes such as LDL/HDL ratio, TG/HDL ratio, and TC/HDL ratio and decreased BMD. To examine (i) the associations of TG and TC and (ii) the associations of TG and LDL-C, either separately or combined, participants were divided into 4 subgroups based on the values of TG and TC as well as TG and LDL-C, respectively. We performed collinearity diagnostics between TG, TC, and HDL-C and between TG, LDL-C, and HDL-C while performing the same test between log TG, TC, and HDL-C, and between log TG, LDL-C, and HDL-C. Since the Variance Inflation Factors among the 3 of them were all below 10, there was no significant collinearity between the 3 variables. Although this was a cross-sectional study, we assumed the exposure as abnormal lipid profiles and outcome as osteopenia or osteoporosis. All statistical operations were performed using the Statistical Package for the Social Sciences (version 25.0; SPSS Inc, Chicago, IL, USA). A *P* value of < .05 was considered significant.

## Results

### The Demographics of 1373 Patients With Available Data for Bone Mineral Density

A total of 1373 subjects were identified, including 793 males and 580 females, with mean age of 61.02 ± 6.83 (mean ± SD) years. Male individuals tended to have a higher height, weight, BMI, WC, DBP, ALT, fasting blood glucose, and uric acid, and higher prevalence of peptic ulcer, while female individuals tended to have higher HDL-C, LDL-C, and TC, and higher prevalence of osteopenia or osteoporosis (Table 1).

**Table 1.** Demographics of the 1373 subjects aged ≥ 50 years with available osteoporosis data

Variables	Total	M	F	*P* value
Number	1373	793	580	
Age, years	61.02 ± 6.83	61.24 ± 6.77	60.72 ± 6.90	.169
Height, cm	163.30 ± 8.23	168.27 ± 5.94	156.52 ± 5.69	<.001
Weight, kg	65.09 ± 11.84	70.65 ± 10.32	57.49 ± 9.28	<.001
BMI, kg/m^2^	24.32 ± 3.50	24.94 ± 3.30	23.47 ± 3.60	<.001
WC, cm	82.77 ± 10.21	86.81 ± 8.92	77.24 ± 9.23	<.001
SBP, mmHg	128.81 ± 19.56	128.68 ± 17.30	129.00 ± 22.27	.769
DBP, mmHg	83.89 ± 10.71	85.98 ± 9.71	81.06 ± 11.35	<.001
Osteopenia or osteoporosis	1075 (78.3%)	573 (72.3%)	502 (86.6%)	<.001
ALT, U/L	30.25 ± 26.80	33.11 ± 28.26	26.35 ± 24.15	<.001
Fasting blood glucose, mg/dL	106.43 ± 28.08	108.50 ± 30.26	103.60 ± 24.53	.001
Uric acid, mg/dL	6.49 ± 1.54	7.01 ± 1.51	5.77 ± 1.28	<.001
HDL-C, mg/dL	51.66 ± 14.00	47.05 ± 12.06	57.97 ± 14.01	<.001
TG, mg/dL	118.56 ± 82.59	127.72 ± 95.86	106.02 ± 57.55	<.001
LDL-C, mg/dL	128.69 ± 34.14	124.54 ± 32.61	134.37 ± 35.37	<.001
TC, mg/dL	209.50 ± 40.05	201.60 ± 38.09	220.32 ± 40.16	<.001
Peptic ulcer	324 (31.4%)	221 (37.0%)	103 (23.7%)	<.001

Abbreviations: ALT, alanine aminotransferase; BMI, body mass index; DBP, diastolic blood pressure; HDL-C, high-density lipoprotein cholesterol, LDL-C, low-density lipoprotein cholesterol; SBP, systolic blood pressure; TC, total cholesterol; TG, triglyceride; WC, waist circumference.

### The Comparison of Clinical Characteristics and the Associated Physical and Laboratory Abnormalities in Patients With Normal Bone Density, Osteopenia, and Osteoporosis

Since female individuals have higher risk of osteopenia or osteoporosis compared to male individuals, we sought other potential confounders for the risk of osteopenia or osteoporosis, so based on the DXA data, the subjects were categorized into 3 groups, including those with normal bone mass density (T-score equal to or more than −1.0, n = 298), those with osteopenia (T-score < −1.0 and greater than −2.5, n = 763), and those with osteoporosis (T-score equal to or less than −2.5, n = 312). In addition to the fact that female patients have higher risk of developing osteoporosis (female vs male, 61.5% vs 38.5%, in the group with osteoporosis), the group with osteoporosis tended to have lower height, weight, BMI, WC, ALT, uric acid, and TG but older age and higher SBP, HDL-C, LDL-C, and TC as compared with the group with normal BMD (Table 2). Taken together, although female patients tended to have osteoporosis, several confounders among the association between osteoporosis and female gender were potentially present, including age, height, weight, BMI, WC, SBP, ALT, uric acid, and lipid profiles.

**Table 2.** The comparison of clinical characteristics and the associated physical and laboratory abnormalities in patients with normal bone density, osteopenia, and osteoporosis

Variables	Normal bone density	Osteopenia*^a^*	Osteoporosis*^b^*	*P* value
Number	298	763	312	
Age, years	59.02 ± 6.16	60.42 ± 6.40	64.38 ± 7.28	<.001
Gender				
F	78 (26.2%)	310 (40.6%)	192 (61.5%)	<.001
M	220 (73.8%)	453 (59.4%)	120 (38.5%)	
Height, cm	167.00 ± 7.38	163.61 ± 7.80	159.03 ± 8.11	<.001
Weight, kg	72.52 ± 12.09	65.16 ± 10.23	57.82 ± 10.79	<.001
BMI, kg/m^2^	25.96 ± 3.88	24.30 ± 3.13	22.79 ± 3.30	<.001
WC, cm	87.07 ± 10.23	82.95 ± 9.38	78.22 ± 10.29	<.001
SBP, mmHg	129.24 ± 18.30	127.71 ± 19.11	131.12 ± 21.58	.039
DBP, mmHg	85.07 ± 10.79	83.83 ± 10.49	82.92 ± 11.11	.056
ALT, U/L	32.26 ± 24.64	30.81 ± 29.09	26.97 ± 22.38	.035
Fasting blood glucose, mg/dL	107.76 ± 25.60	106.66 ± 30.29	104.61 ± 24.49	.362
Uric acid, mg/dL	6.88 ± 1.40	6.51 ± 1.55	6.07 ± 1.56	<.001
HDL-C, mg/dL	47.82 ± 12.62	51.16 ± 13.42	56.55 ± 15.25	<.001
TG, mg/dL	123.67 ± 75.42	122.74 ± 90.76	103.52 ± 64.71	.002
LDL-C, mg/dL	124.27 ± 32.31	130.25 ± 34.84	129.09 ± 33.86	.042
TC, mg/dL	201.17 ± 36.89	211.25 ± 40.52	213.16 ± 40.80	<.001
Peptic ulcer	73 (36.7%)	174 (30.6%)	77 (29.2%)	.186

Abbreviations: ALT, alanine aminotransferase; BMI, body mass index; DBP, diastolic blood pressure; HDL-C, high-density lipoprotein cholesterol, LDL-C, low-density lipoprotein cholesterol; SBP, systolic blood pressure; TC, total cholesterol; TG, triglyceride; WC, waist circumference.

^*a*^Osteopenia: −2.5 < T-score < −1.

^*b*^Osteoporosis: T-score ≤ −2.5.

### Subgroup Analysis Stratified by Gender With Multiple Logistic Regression Showed That Patients With TC ≥ 240 mg/dL, or LDL-C ≥ 160 mg/dL Had Significantly Increased Association With Osteoporosis or Osteopenia

To examine the association between LDL-C, TG, and TC with decreased BMD, we performed subgroup analysis stratified by gender with multiple logistic regression. We categorized the subjects into 3 groups according to TG level as (i) TG < 150 mg/dL; (ii) TG: 150∼199 mg/dL; and (iii) TG ≥ 200 mg/dL (Table 3). Compared to people with TG< 150 mg/dL, those with TG ≥ 200 mg/dL had higher association with the presence of osteopenia or osteoporosis (odds ratio [OR] 1.86; 95% CI, 0.98-3.52) after adjustment for age, gender, BMI, SBP, DBP, fasting blood glucose, uric acid, HDL-C, ALT, and peptic ulcer, although this was statistically insignificant. Similarly, we also divided subjects into 3 groups according to TC level as (i) TC < 200 mg/dL; (ii) TC: 200∼239 mg/dL; and (iii) TC ≥ 240 mg/dL. Compared to people with TC < 200 mg/dL, those with TC ≥ 240 mg/dL had higher association with the presence of osteopenia or osteoporosis (OR 2.61; 95% CI, 1.44-4.71) after adjustment for the additional covariates. Finally, we divided subjects into 3 groups according to LDL-C level as (i) LDL-C < 130 mg/dL; (ii) LDL-C: 130∼159 mg/dL; and (iii) LDL-C ≥ 160 mg/dL. Compared to people with LDL-C < 130 mg/dL, those with LDL-C ≥ 160 mg/dL had higher association with osteopenia or osteoporosis (OR 2.13; 95% CI, 1.21-3.74) after adjustment for the same covariates (Table 3). We also performed subgroup analysis as stratified by gender, and in male subjects, compared to people with TC < 200 mg/dL, those with TC ≥ 240 mg/dL showed significant increase in association with osteopenia or osteoporosis (OR 2.42; 95% CI, 1.18-4.98) after adjustment for the additional covariates. Compared to people with LDL-C < 130 mg/dL, those with LDL-C ≥ 160 mg/dL revealed significant increase in association with osteopenia or osteoporosis (OR 2.28; 95% CI, 1.11-4.68) after adjustment for the same covariates (Table 3). In females, compared to people with TG < 150 mg/dL, TC < 200 mg/dL, and LDL-C < 130 mg/dL, those with TG > 200 mg/dL, TC > 240 mg/dL, and LDL-C > 160 mg/dL had increased associations with decreased BMD, although these were not statistically significant ([TG] OR 3.30; 95% CI, 0.39-27.87; [TC] OR 2.34; 95% CI, 0.77-7.14; and [LDL-C] OR 1.87; 95% CI, 0.72-4.86).

**Table 3.** Relationship between lipid profile and osteopenia or osteoporosis in simple and multiple logistic regression stratified by gender

Variables (mg/dL)		Total	Normal BMD	Decreased BMD	OR (95% CI)	aOR (95% CI)*^a^*	aOR (95% CI)*^b^*
TG	<150	1047	212 (20.20)	835 (79.80)	ref	ref	ref
	150-199	187	48 (25.70)	139 (74.30)	0.74 (0.51,1.06)	1.18 (0.79,1.74)	1.64 (0.96,2.81)
	≥200	136	37 (27.20)	99 (72.80)	0.68 (0.45,1.02)	1.32 (0.85,2.05)	1.86 (0.98,3.52)
TC	<200	568	138 (24.30)	430 (75.70)	ref	ref	ref
	200-239	515	124 (24.10)	391 (75.90)	1.01 (0.77,1.34)	0.97 (0.72,1.31)	0.97 (0.66,1.41)
	≥240	287	35 (12.20)	252 (87.80)	2.31*** (1.55, 3.46)	2.22*** (1.45, 3.41)	2.61** (1.44, 4.71)
LDL-C	<130	730	170 (23.30)	560 (76.70)	ref	ref	ref
	130-159	405	93 (23.0)	312 (77.0)	1.02 (0.76-1.34)	1.15 (0.85-1.57)	1.06 (0.72-1.55)
	≥160	234	34 (14.50)	200 (85.50)	1.79** (1.20-2.67)	1.78** (1.17-2.73)	2.13** (1.21-3.74)
**Female**		Total	normal BMD	decreased BMD	OR (95% CI)	aOR (95% CI)*^c^*	aOR (95% CI)*^d^*
TG	<150	472	61 (12.90)	411 (87.10)	ref	ref	ref
	150-199	70	12 (17.10)	58 (82.90)	0.72 (0.36-1.41)	1.12 (0.49-2.55)	1.71 (0.50-5.82)
	≥200	36	4 (11.10)	32 (88.90)	1.19 (0.41-3.47)	2.42 (0.68-8.56)	3.30 (0.39-27.87)
TC	<200	174	26 (14.90)	148 (85.10)	ref	ref	ref
	200-239	233	38 (16.30)	195 (83.70)	0.90 (0.52-1.55)	1.18 (0.65-2.16)	0.68 (0.29-1.61)
	≥240	171	13 (7.60)	158 (92.40)	2.14* (1.06-4.31)	2.85** (1.31-6.22)	2.34 (0.77-7.14)
LDL-C	<130	278	44 (15.80)	234 (84.20)	ref	ref	ref
	130-159	171	19 (11.10)	152 (88.90)	1.50 (0.85-2.68)	2.10* (1.07-4.10)	1.48 (0.62-3.56)
	≥160	129	14 (10.90)	115 (89.10)	1.55 (0.81-2.93)	1.84 (0.90-3.78)	1.87 (0.72-4.86)
**Male**		Total	normal BMD	decreased BMD	OR (95% CI)	aOR (95% CI)*^c^*	aOR (95% CI)*^d^*
TG	<150	575	151 (26.30)	424 (73.70)	ref	ref	ref
	150-199	117	36 (30.80)	81 (69.20)	0.80 (0.52-1.24)	1.13 (0.71-1.78)	1.57 (0.85-2.89)
	≥200	100	33 (33.00)	67 (67.00)	0.72 (0.46-1.14)	1.06 (0.66-1.72)	1.57 (0.79-3.11)
TC	<200	394	112 (28.40)	282 (71.60)	ref	ref	ref
	200-239	282	86 (30.50)	196 (69.50)	0.91 (0.65-1.27)	0.91 (0.64-1.29)	1.00 (0.65-1.54)
	≥240	116	22 (19.00)	94 (81.00)	1.70* (1.02-2.84)	1.92* (1.13-3.26)	2.42* (1.18-4.98)
LDL-C	<130	452	126 (27.90)	326 (72.10)	ref	ref	ref
	130-159	234	74 (31.60)	160 (68.40)	0.84 (0.59-1.18)	0.93 (0.65-1.32)	0.94 (0.60-1.46)
	≥160	105	20 (19.00)	85 (81.00)	1.64 (0.97-2.79)	1.74* (1.01-3.00)	2.28* (1.11-4.68)

Abbreviations: BMD, bone mineral density; LDL-C, low-density lipoprotein cholesterol; TC, total cholesterol; TG, triglyceride.

^*a*^Adjusted for age, gender, body mass index (BMI).

^*b*^Adjusted for age, gender, BMI, systolic blood pressure (SBP), diastolic blood pressure (DBP), fasting blood glucose, uric acid, high-density lipoprotein cholesterol (HDL-C), alanine aminotransferase (ALT), peptic ulcer.

^*c*^Adjusted for age, BMI.

^*d*^Adjusted for age, BMI, SBP, DBP, fasting blood glucose, uric acid, HDL-C, ALT, peptic ulcer.

*P < .05, **P < .01, ***P < .001.

In short, a significant increase in association with osteopenia or osteoporosis in patients with elevated TC (≥ 240 mg/dL) and LDL-C (≥ 160 mg/dL) was found after adjustment of gender, age, BMI, and other confounders (Table 3).

### Association Between Atherogenic Index and Osteopenia or Osteoporosis in Simple and Multiple Logistic Regression Stratified by Gender

Since higher levels of TC or LDL-C were found to be associated with osteopenia or osteoporosis, we sought to determine whether atherogenic indexes such as TG/HDL, TC/HDL, or LDL-C/HDL would produce additional increase in the association with low BMD. By multiple logistic regression to adjust potential confounders, we found that increases in TC/HDL were marginally associated with osteopenia or osteoporosis (OR 1.17; 95% CI, 1.001-1.37). However, this marginal statistical significance does not necessarily confer a true clinical impact. In subgroup analysis stratified by gender, increases in TG/HDL, TC/HDL, or LDL-C/HDL were associated with low BMD in men and women but these associations were not statistically significant (Table 4).

**Table 4.** Relationship between atherogenic index and osteopenia or osteoporosis in simple and multiple logistic regression stratified by gender

Variables	OR (95% CI)	aOR (95% CI)*^a^*	aOR (95% CI)*^b^*
TG/HDL	0.95* (0.90-0.99)	1.05 (0.99-1.12)	1.06 (0.98-1.16)
TC/HDL	0.87* (0.78-0.97)	1.10 (0.97-1.24)	1.17* (1.001-1.37)
LDL-C/HDL	0.86* (0.75-0.98)	1.09 (0.93-1.26)	1.19 (0.98-1.44)
**Female**	OR (95% CI)	aOR (95% CI)*^c^*	aOR (95% CI)*^d^*
TG/HDL	0.94 (0.81-1.09)	1.14 (0.93-1.41)	1.06 (0.79-1.42)
TC/HDL	0.99 (0.79-1.24)	1.31 (0.97-1.76)	1.26 (0.85-1.89)
LDL-C/HDL	0.99 (0.76-1.30)	1.35 (0.95-1.92)	1.30 (0.81-2.08)
**Male**	OR (95% CI)	aOR (95% CI)*^c^*	aOR (95% CI)*^d^*
TG/HDL	0.98 (0.93-1.03)	1.03 (0.97-1.10)	1.05 (0.96-1.15)
TC/HDL	0.91 (0.80-1.04)	1.02 (0.89-1.17)	1.12 (0.94-1.33)
LDL-C/HDL	0.90 (0.76-1.05)	0.99 (0.84-1.17)	1.13 (0.92-1.40)

Abbreviations: aOR, adjusted odds ratio; HDL-C, high-density lipoprotein cholesterol, LDL-C, low-density lipoprotein cholesterol; OR, odds ratio; TC, total cholesterol; TG, triglyceride.

^*a*^Adjusted for age, gender, body mass index (BMI).

^*b*^Adjusted for age, gender, BMI, systolic blood pressure (SBP), diastolic blood pressure (DBP), fasting blood glucose, uric acid, alanine aminotransferase (ALT), peptic ulcer.

^*c*^Adjusted for age, BMI.

^*d*^Adjusted for age, BMI, SBP, DBP, fasting blood glucose, uric acid, ALT, peptic ulcer.

*P < .05, **P < .01, ***P < .001.

### Combined Elevated Blood Levels of TG (≥200 mg/dL) and TC (≥240 mg/dL) Were Significantly Associated With Osteoporosis or Osteopenia

Since we found that higher TC or LDL-C were associated with osteopenia or osteoporosis, we sought to determine whether elevated TG and elevated TC revealed higher association with low BMD. Using multiple logistic regression to adjust potential confounders, we found that those with TG < 200 mg/dL and TC ≥ 240 mg/dL might produce higher association with the presence of osteopenia or osteoporosis (OR 2.50; 95% CI, 1.34-4.65) after adjustment for the additional covariates. People with TG ≥ 200 mg/dL and TC ≥ 240 mg/dL also might have higher association with suffering from osteopenia or osteoporosis (OR 3.51; 95% CI, 1.11-11.13) after adjustment for the same covariates, compared to subjects who had TG < 200 mg/dL and TC < 240 mg/dL (Table 5).

**Table 5.** Relationship between lipid profile subgroup classification (TG/TC, TG/LDL-C) and osteopenia or osteoporosis in simple and multiple logistic regression

TG (mg/dL)	TC (mg/dL)	Total	Normal BMD	Decreased BMD	OR (95% CI)	aOR (95% CI)*^a^*	aOR (95% CI)*^b^*
<200	<240	992	232 (23.40)	760 (76.60)	ref	ref	ref
≥200	<240	91	30 (33.0)	61 (67.0)	0.62* (0.39, 0.98)	0.99 (0.60, 1.62)	1.30 (0.64, 2.64)
<200	≥240	242	28 (11.60)	214 (88.40)	2.33*** (1.53, 3.55)	2.03** (1.30, 3.17)	2.50** (1.34, 4.65)
≥200	≥240	45	7 (15.60)	38 (84.40)	1.66 (0.73, 3.76)	3.18** (1.37, 7.40)	3.51* (1.11, 11.13)

TG (mg/dL)	LDL-C (mg/dL)	Total	Normal BMD	Decreased BMD	OR (95% CI)	aOR (95% CI)*^a^*	aOR (95% CI)*^b^*
<200	<160	1028	230 (22.40)	798 (77.60)	ref	ref	ref
≥200	<160	107	33 (30.80)	74 (69.20)	0.65 (0.42, 1.00)	1.10 (0.69, 1.75)	1.34 (0.69, 2.59)
<200	≥160	205	30 (14.60)	175 (85.40)	1.68* (1.11,2.54)	1.51 (0.98, 2.34)	1.81* (1.03, 3.18)
≥200	≥160	29	4 (13.8)	25 (86.20)	1.80 (0.62, 5.23)	3.35* (1.12, 9.98)	9.31* (1.15, 75.42)

Abbreviations: aOR, adjusted odds ratio; BMD, bone mineral density; LDL-C, low-density lipoprotein cholesterol; OR, odds ratio; TC, total cholesterol; TG, triglyceride.

^*a*^Adjusted for age, gender, body mass index (BMI).

^*b*^Adjusted for age, gender, BMI, systolic blood pressure (SBP), diastolic blood pressure (DBP), fasting blood glucose, uric acid, high-density lipoprotein cholesterol (HDL-C), alanine aminotransferase (ALT), peptic ulcer.

*P < .05, **P < .01, ***P < .001.

### The Combined Presence of Elevated Blood Levels of TG (≥200 mg/dL) and LDL-C (≥160 mg/dL) Was Significantly Associated With Osteoporosis or Osteopenia

Because we found that elevated TG and elevated TC had higher association with low BMD, we investigated whether elevated TG and elevated LDL-C had a more profound association with low BMD. With multiple logistic regression, it was found that those with TG < 200 mg/dL and LDL-C ≥ 160 mg/dL might have higher association with having osteopenia or osteoporosis (OR 1.81; 95% CI, 1.03-3.18) after adjustment for the additional covariates. People with TG ≥ 200 mg/dL and LDL-C ≥ 160 mg/dL might have very high association with osteopenia or osteoporosis (OR 9.31; 95% CI, 1.15-75.42) after adjustment for the same covariates, compared to subjects who had TG < 200 mg/dL and LDL-C < 160 mg/dL (Table 5).

## Discussion

In this cross-sectional study of adults with age ≥ 50 years, we found TC, LDL-C, and TG to be associated with osteopenia or osteoporosis. After adjusting for age, BMI, and other covariates, individuals with increased TC (≥ 240 mg/dL) or LDL-C (≥ 160 mg/dL) had an increased risk of osteopenia or osteoporosis, especially in men. We also found that increased atherogenic index (TC/HDL ratio) was associated with decreased BMD. After adjusting for the same variables, individuals with TG ≥ 200 mg/dL and TC ≥ 240 mg/dL also showed a significantly increased association with osteopenia or osteoporosis (OR 3.51; 95% CI, 1.11-11.13) as compared to patients with TG < 200 mg/dL and TC < 240 mg/dL. After adjusting for the same variables, those with TG ≥ 200 mg/dL and LDL-C ≥ 160 mg/dL exhibited very significant association with osteopenia or osteoporosis (OR 9.31; 95% CI, 1.15-75.42) as compared to those with TG < 200 mg/dL and LDL-C < 160 mg/dL.

One previous study has also reported that higher TC and TG levels were associated with a greater risk of osteoporosis [20]; however, that study focused on osteoporosis alone while ours included both osteopenia and osteoporosis in the study outcome, and the effects of TC, LDL-C, and TG, both individually and in combination, were not compared in the previous study. We found the combination of 2 abnormal lipid profiles had a higher association with low bone density in this research.

The significant associations between TC, TG, and LDL-C levels, and osteoporosis in our study are consistent with those in earlier research studies [19, 20, 26] that showed a positive association between lipid profiles and osteoporosis. We found no significant links between HDL-C and osteoporosis. This finding is in part compatible with those of Ghadiri-Anari et al [18], who discovered no significant associations between HDL-C and BMD. However, previous studies of the relationship between BMD and serum lipid profiles revealed conflicting results. Samelson et al examined blood cholesterol in 712 women and 450 men who were enrolled in the Framingham study [27], although bone density was not tested until 34 years later (1988-1989), finding that cholesterol levels in women and men from early adulthood to middle age did not appear to have long-term clinical implications for osteoporosis later in life. In a cross-sectional study conducted in China, higher serum HDL-C levels were associated with a higher risk of osteoporosis than lower HDL-C levels, while TC, TG, and LDL-C were not associated with BMD [16]. A recent meta-analysis that examined the relationship between lipid profiles and osteoporosis in postmenopausal women revealed that TC and HDL-C levels were statistically significantly higher in postmenopausal women with osteoporosis than in the control group [22]. Different research sample sizes, study designs, populations, and factors adjusted for in the study are likely to attribute to these disparate outcomes.

In this study, we found that individuals with TC > 240 mg/dL and LDL-C > 160 mg/dL had a stronger association with lower bone density in men but not in women, as compared to those with TC < 200 mg/dL and LDL-C < 130 mg/dL. Only a few studies have investigated the gender effect on association of lipid profiles and decreased bone density [5, 23]. Women experience bone loss in 2 stages: the first starts at menopause and mostly affects the trabecular bone [28]. In this phase, osteoporosis results from lack of estrogen that causes a disproportionate increase in bone resorption relative to bone growth. One could characterize this stage as bone loss along with menopause. Following 4 to 8 years, the second phase, so-called age-related bone loss, is characterized by a persistent, slower loss of cortical and trabecular bone, which is primarily caused by a decrease in bone synthesis. The second phase, but not the first phase, also affects men [28]. That we did not find statistically significant associations between abnormal lipid profiles and decreased BMD in women might have resulted from several factors, including the smaller sample size for women, fewer female subjects had a smoking habit, and the lack of data on estrogen level and precise menopausal age.

In our study, we did not find a significant association between fasting blood sugar and decreased bone density, which could result from most of our subjects being healthy or under good sugar control. Previous studies have indicated that type 2 diabetes and metabolic syndrome may be associated with decreased bone density, but the association remains debatable [23, 29, 30]. For example, Isaia et al found that patients with type 2 diabetes had lower BMD [29] where bone mass due to impaired metabolic regulation occurs before diabetes was diagnosed. On the other hand, Tseng et al found no significant association between fasting glucose and BMD [30]. Loke et al discovered that although metabolic syndrome (having ≥ 3 components of metabolic syndrome) was inversely linked with BMD in women, it was positively associated with BMD in men [23]. The detailed mechanism for the gender difference in the risk of osteoporosis, and the causal association between BMD with metabolic syndrome are unknown and might be clarified by a prospective study. We were not able to perform analysis for the association between metabolic syndrome and decreased BMD due to lack of data of drug history for hypertension and diabetes.

The positive associations of TC, LDL-C, and TG levels with decreased bone density might be explained by several biological pathways. On a molecular and cellular level, bone and vascular tissue have several similarities; for example, endothelial cells, pre-osteoblasts, and monocyte-derived osteoclasts are all found in bone marrow, and they all have counterparts in the arterial wall [31]. Atherosclerosis and osteoporosis are associated with oxidized lipids, which have distinct effects on the arterial wall and bone; for example, the oxidized lipids cause osteoblastic cells in the arterial wall to mineralize and differentiate, leading to atherosclerosis. In contrast, in bone and bone osteoblasts, oxidized lipids can suppress osteoblastic cell differentiation and inhibit bone cell growth while lowering BMD [12-14]. Lipids in the blood can also impact osteoclasts in bone [31] where the oxidation and metabolism of lipids and lipoproteins in bone tissues can cause osteoclast development, which leads to bone tissue loss [32, 33].

At the molecular level, by inhibiting alkaline phosphatase activity, extracellular matrix maturation, and mineralization, oxidized lipids can promote inflammatory response in bone and block osteoblast differentiation [15, 34, 35]. By enhancing tartrate-resistant acid phosphatase (TRAP) activity, which is related to the development of multinucleated cells and mineral resorption, oxidized lipids can activate osteoclasts via a cAMP-mediated mechanism that triggers RANKL-dependent osteoclastic differentiation of these cells [36, 37]. In both in vitro and in vivo, oxidized lipids were found to induce the expression of cytokines such as monocyte chemoattractant protein 1 (MCP-1), macrophage colony-stimulating factor (M-CSF), and interleukin-6 [38, 39]. The nuclear hormone receptor peroxisome proliferator activated receptor ɣ (PPAR-ɣ) may have a role in the relationship between lipid biomarkers and BMD, according to the research that has shown that lipid metabolites can activate the PPAR-ɣ and osteogenesis is hindered when PPAR-ɣ levels rise [40, 41].

Numerous observational cohort or case-control studies in humans have revealed that statin users had a reduced incidence of fractures or higher BMD than nonusers [42, 43], although several investigations have reported contradictory results [44, 45]. In one Taiwanese cohort research, statin usage was linked to a lower incidence of osteoporosis in both men and women, with the osteoprotective impact of statins being more significant with a reliance on cumulative dosage and statin intensity [46]. Statins have been found to reduce TC and TG levels and the risk of osteoporosis in patients, which is another finding that supports this current study.

This study has several limitations. Firstly, the subjects were not randomly selected, so there may have been some selection bias, and second, the individuals’ lifestyle characteristics (such as smoking, alcohol use, and physical activity), drug history (proton pump inhibitor usage, calcium intake), and vitamin D levels were not considered due to a lack of data. Third, data on renal function were lacking. Although osteoporosis, fragility fractures, and mineral bone disorder are all associated with chronic kidney disease [47], the majority of our participants were healthy adults so the prevalence of chronic kidney disease might not have been as high as that in case-control studies.

Fourth, Table 5 shows only a few subjects in some of the groups; however, we repeated the analysis, for which the results were consistent. Finally, the findings were derived from a cross-sectional study, so causal inference about the association between lipid biomarkers and BMD might exist. A prospective cohort study could further clarify the cause-and-effect link between BMD and lipid profiles.

In conclusion, through this retrospective cross-sectional study from 1373 patients, we found that having one abnormal lipid profile such as a high TC (≥ 240 mg/dL), or a high LDL-C (≥ 160 mg/dL), was positively associated with decreased BMD, especially in men. Moreover, an increase in the number of abnormal lipid profiles such as high TC (≥240 mg/dL) and high TG (≥200 mg/dL), or high LDL-C (≥160 mg/dL) and high TG (≥200 mg/dL), had significantly increased positive association with decreased BMD. Based on this study, we recommend that individuals aged more than 50 years with abnormal lipid profiles or with increased atherogenic index (TC/HDL ratio) be eligible for a bone density survey to exclude osteopenia or osteoporosis. Controlling blood lipid profiles might prevent not only cardiovascular diseases but osteoporosis as well.

## Data Availability

Restrictions apply to the availability of some or all data generated or analyzed during this study to preserve patient confidentiality or because they were used under license. The corresponding author will on request detail the restrictions and any conditions under which access to some data may be provided.

## Abbreviations

ALT: alanine aminotransferase
BMD: bone mineral density
BMI: body mass index
DBP: diastolic blood pressure
DXA: dual-energy x-ray absorptiometry
HDL-C: high-density lipoprotein cholesterol
LDL-C: low-density lipoprotein cholesterol
OR: odds ratio
SBP: systolic blood pressure
TC: total cholesterol
TG: triglyceride
WC: waist circumference
